# 

**Published:** 1891-09

**Authors:** 


### LITERARY.

### A NEW STANDARD AMERICAN DICTIONARY.

The production of a complete dictionary of the language of a people, is a great undertakIng. It is especially so in respect to the English, in the face of the two excellent ones of Webster and Wooster, which it will be found no easy matter to surpass. Nevertheless the enterprising publishers, Funk & Wagnall, 18 and 20 Astor Place, New York City, have undertaken the herculean task, and have it, even now, well under way. The roll of over a hundred eminent scholars, at present enlisted as editors of the work, is a sure guarantee of its completeness, and we doubt not it is destined to take its place in the front rank, if not altogether in the lead of American Lexicologies, which for many years have maintained the first place with all English speaking races.

---

### THE TENNESSEE MEDICAL COLLEGE.

Located at Knóxville, makes a handsome showing the present year. Matriculants, 67. Graduates in Medicine, 18. In Dentistry, 7. The Lecture rooms, Laboratories, are very complete, and all the best means of a thorough medical education under the guidance of an excellent faculty.

---

THE SOUL is the reality of our existence. To speak accurately the human visage is a mask. The true man is that which exists under what is called man, if that being which thus exists sheltered and screened behind that illusion could be approached, more than one strange revelation would be made. The vulgar error is to mistake the outward husk for the living spirit.—VICTOR HUGO.

